# Efficacy of Continuous Renal Replacement Therapy and Intermittent Hemodialysis in Patients with Renal Failure in Intensive Care Unit: A Systemic Review and Meta-analysis

**DOI:** 10.1155/2023/8688974

**Published:** 2023-04-17

**Authors:** Haiying Ma, Hengjian Liu, Yi Liu, Yi Wang, Jiang He, Qiaoyun Yang

**Affiliations:** ^1^Department of Critical Care Medicine, Changzhou Hospital of Traditional Chinese Medicine, Changzhou, Jiangsu, China; ^2^Department of Critical Care Medicine, Changzhou No. 2 People's Hospital, Changzhou, Jiangsu, China

## Abstract

**Objective:**

This study aimed to compare the clinical efficacy of continuous renal replacement therapy (CRRT) and intermittent hemodialysis (IHD) in patients with renal failure in intensive care unit (ICU).

**Methods:**

Relevant studies were searched in the databases including EMBASE, Cochrane Library, and MEDLINE (PubMed) from inception to January 04, 2021. The inclusion of available studies and the collection of data were independently conducted by two authors after reviewing the full text. Pooled analyses of relative risk (RR) and weighted mean difference (WMD) were performed to compare the outcomes of renal recovery, short-term mortality, length of ICU stays, and length of in-hospital stays between the two different treatment groups. Publication bias was assessed by the funnel plot.

**Results:**

A total of 11 RCT studies including 1740 patients with renal failure were eligible for final analysis. Among them, 894 patients (51.4%) underwent CRRT and 846 patients (48.6%) received IHD. Pooled analysis did not find significant differences in renal recovery and short-term mortality between the two groups. Interestingly, patients underwent CRRT showed significantly shorter length of ICU stay and in-hospital stay than those who underwent IHD (ICU stay: RR: −0.61, 95%CI: −1.10—−0.11, *P* < 0.05; I^2^ = 93.6%; in-hospital stay: RR: −0.56, 95%CI: −1.41–0.28, *P* < 0.05; I^2^ = 97.7%). No significant publication biases were observed on the funnel plots.

**Conclusion:**

Compared with IHD, CRRT had similar effects on renal recovery and short-term mortality in patients with renal failure in ICU. As a promising technique in clinical practice, CRRT could significantly reduce the length of ICU stay and in-hospital stay of patients, which was of great significance for the reduction of medical costs and the long-term benefits of patients, thereby reducing the burden on society and individuals.

## 1. Introduction

Acute kidney injury (AKI) is a clinical syndrome characterized by an acute decrease in glomerular filtration rate [1–3]. Mild patients with AKI have no obvious symptoms and recover quickly, whereas severe acute patients have a higher mortality rate [4]. Due to the reduced glomerular filtration rate, metabolic wastes cannot be discharged in time, which will affect the balance of water and electrolytes in body. In severe cases, kidney injury can lead to multiple organ failure, endangering the life of the patient. The incidence of AKI in general population was only 5%, and it was significantly higher in intensive care unit (ICU) patients, with an incidence of 36-70% and a mortality rate of 40-70% [5]. In China, the number of AKI patients reached 2.9 million in 2013, of which 700,000 died, and the treatment cost was 13 billion US dollars [6], causing a huge disease burden to the society.

At present, one of the effective clinical treatments for AKI is blood purification, among which intermittent hemodialysis (IHD) and continuous renal replacement therapy (CRRT) are more commonly used [7]. IHD adopts the principle of diffuse convection to remove harmful substances such as inflammatory mediators and endogenous and exogenous toxins in the blood of patients through dialysis equipment, which has a certain effect on improving the patient's condition [8]. However, due to the characteristics of significant changes in the composition of dialysate, blood gas parameters, and blood membrane reaction, patients are prone to hypotension after solute and water are removed in a short period of time. Meanwhile, the removal of small molecular substances such as norepinephrine and epinephrine will aggravate the hypotension, which has a serious impact on the recovery of renal function, resulting in decreased survival [9].

In recent years, with the development of medical technology, CRRT has been widely used in the treatment of patients with severe acute renal failure [10]. Similar to IHD, CRRT removes inflammatory mediators mainly by convection and adsorption, and it continuously and slowly removes water and solutes in the body [11]. The major advantage of CRRT is the stable hemodynamics. Because dehydration is slow, the incidence of hypotension and arrhythmia is low, and there is no renal ischemia, which is conducive to the recovery of renal function. Currently, there is still controversy about the selection of treatment strategy for the treatment of patients with AKI in ICU. Identifying the best treatment for renal impairment in patients with renal failure in ICU and clarifying the specific advantages of various treatment options are important to effectively improve patient outcomes and reduce the economic burdens of social and families.

Herein, we conducted this systemic review and meta-analysis aimed to compare the clinical efficacy of CRRT and IHD in patients with renal failure in ICU and further provided evidence for helping clinicians to make a better treatment strategy.

## 2. Materials and Methods

### 2.1. Search of Studies

We performed the systemic review and meta-analysis by strictly following the instructions and requirements in the Preferred Reporting Items for Systematic Reviews and Meta-Analyses (PRISMA) [12]. The key words “continuous renal replacement therapy” and “intensive care units” and “kidney failure, chronic” or “renal insufficiency” or “acute kidney injury” and “randomized controlled trial” were used for searching the relevant studies in the databases including Cochrane Library, EMBASE, and MEDLINE (PubMed) from inception to January 04, 2021. Two authors independently conducted the literature search. First, the potentially relevant literature studies were retrieved from the abovementioned databases; second, these literature studies were screened by reviewing the tittle and abstract; third, the studies meeting the inclusion/exclusion criteria were eventually included after carefully reading the full-text. If there were discrepancies on the inclusion/exclusion of a certain study, a senior researcher was consulted and the study was determined by comprehensive discussion.

### 2.2. Inclusion and Exclusion of Studies

Studies meeting all the following inclusion criteria were eligible: (1) patients included were those with renal failure in the ICU; (2) type of studies were randomized controlled study (RCT); (3) studies directly comparing CRRT modalities to IHD modalities; and (4) studies with ethic statement and signed informed consent forms.

The following studies were excluded: (1) included patients had undergone hemodialysis before admitting to the ICU; (2) included patients with acute drug poisoning; (3) studies with incomplete result data; (4) studies published in the form of reviews, case reports, systemic reviews, and conference abstract.

### 2.3. Extraction of Data

After collecting all the eligible studies, the relevant data were extracted and recorded by the abovementioned two investigators. Any inconsistencies were resolved by consensus. Data for study identifier, the author, year of publication, country, study design, setting, sample size, mean age, proportion of males, other population characteristics, disease diagnosis, primary outcomes, secondary outcomes, risks, ethical approval, study limitations, and other important information were collected with a predesigned form.

### 2.4. Outcomes

The primary outcome was the recovery of kidney function. The secondary outcomes included numbers of patients free of CRRT after discontinuing CRRT, days in ICU, days in hospital, mortality, serum creatinine (SCr), blood urea nitrogen (BUN), and hemodynamic index.

### 2.5. Evaluation of Bias and Study Quality

All the eligible studies were RCTs, so the Revised Cochrane Risk of Bias Tool (RoB2.0) was used for assessing the risk of bias and quality of each study. The publication bias was evaluated by drawing funnel plots and visually assessing the symmetry of the plots. The quality of studies and evaluation of bias were shown in supplementary Figure 1 for details.

### 2.6. Statistical Analyses

Data were analyzed by Stata 14.0, and the pooled analysis was performed by Review Manager 5.0. For studies with binary variables and continuous variables, we collected the relative risk (RR) and standard mean difference (SMD). The between-study heterogeneity was evaluated by the Cochran *Q* test and I^2^ statistic. A significant heterogeneity was indicated by a *P* < 0.01 in *Q* test or I^2^ statistic >50%. In the meta-analysis, the random-effect model was adopted in the analysis of quantitative data. If I^2^ statistic ≤50% or *P* ≥ 0.01 in *Q* test, it was indicated no significant heterogeneity, and the fixed-effect model was used for pooled analysis.

## 3. Results

### 3.1. Inclusion of Studies

After searching the literature studies in the abovementioned databases with the key words, a total of 811 articles were retrieved. Among them, 128 articles were duplicated literature and were excluded. By reviewing the title, 41 relevant articles were obtained. From these articles, 18 literature studies were excluded after carefully reading the abstract. Eventually, a total of 11 articles [13–23] were included since 12 articles did not meet the inclusion criteria (6 articles were cohort studies or reviews; 3 studies were lack of control group; 1 article with unavailable data; and 2 articles with the same study population or from the same institution).

A total of 11 RCT studies with 1740 patients with renal failure were eventually included. Among them, 894 patients (51.4%) underwent CRRT and 846 patients (48.6%) received IHD. As for the gender composition ratio, there were 1026 males (62.7%) and 610 females (37.3%). In terms of the CRRT in these 11 studies, continuous venous-venous hemodialyses (CVVH) were used in 9 studies, continuous arterial-venous hemodialysis was used in 1 study, and continuous hemodialysis filtration was used in 1 study. The characteristics of included studies are shown in Table 1 for details.

### 3.2. Comparison of the Clinical Efficacy between CRRT and IHD in Patients with Renal Failure

#### 3.2.1. Renal Recovery

**Figure 1:**
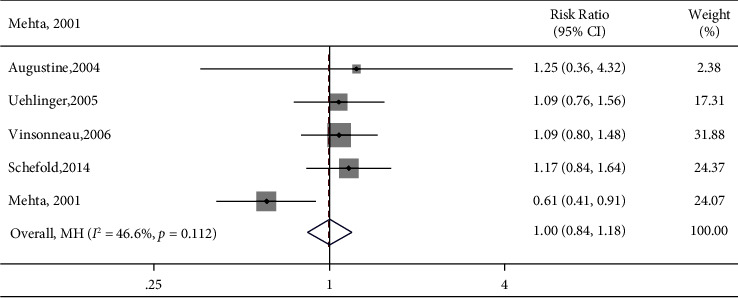
Forest plot of risk ratio and 95% CI of renal recovery. 95% CI: 95% confidence interval. Note: Weights are from Mantel-Haenszel model.

A total of 5 studies evaluated the efficacy of CRRT and IHD on renal recovery at the time of discharge in patients with renal failure [13, 18, 20–22]. Meta-analysis showed that 170 and 176 patients obtained renal recovery after the CRRT and IHD, respectively. Pooled analysis did not find significant differences in renal recovery between the two treatment strategies (RR: 1.00, 95% CI: 0.84-1.18, *P*=0.112; I^2^ = 46.6%, Figure 1). The funnel plot did not show significant asymmetry (Supplementary Figure 2), indicating that there was no significant publication bias of these studies.

#### 3.2.2. Short-Term Mortality

**Figure 2:**
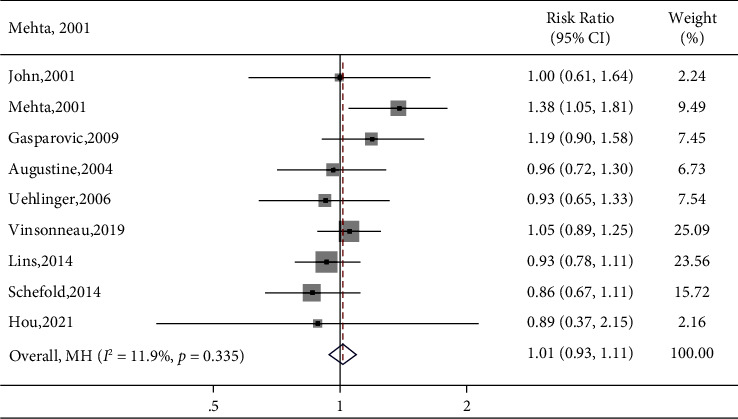
Forest plot of risk ratio and 95% CI of short-term mortality. 95% CI: 95% confidence interval. Note: Weights are from Mantel-Haenszel model.

After systemic review, we found that 9 studies [13–18, 20–22] assessed the short-term mortality of patients after CRRT or IHD. Interestingly, no significant difference was detected between short-term mortality between patients receiving CRRT and IHD (RR: 1.01, 95% CI: 0.93-1.11, *P*=0.335; I^2^ = 11.9%, Figure 2). The funnel plot did not show significant publication bias (Supplementary Figure 3).

#### 3.2.3. Length of ICU Stay

**Figure 3:**
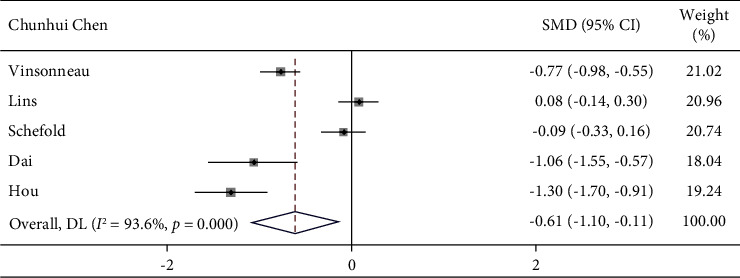
Forest plot of standardized mean difference and 95% CI of length of ICU stay. SMD: standardized mean difference; 95% CI: 95% confidence interval; ICU: intensive care unit. Note: Weights are from random-effects model.

In order to further compare the efficacy of CRRT and IHD in patients with renal failure in ICU, we conducted a pooled analysis of the length of ICU stay of these patients. The results of 5 included studies showed that CRRT significantly shortened the length of ICU stay of patients compared with that of IHD (RR: −0.61, 95% CI: −1.10-−0.11, *P* < 0.05; I^2^ = 93.6%, Figure 3). No significant publication bias was observed on the funnel plot (Supplementary Figure 4).

#### 3.2.4. Length of In-Hospital Stay

**Figure 4:**
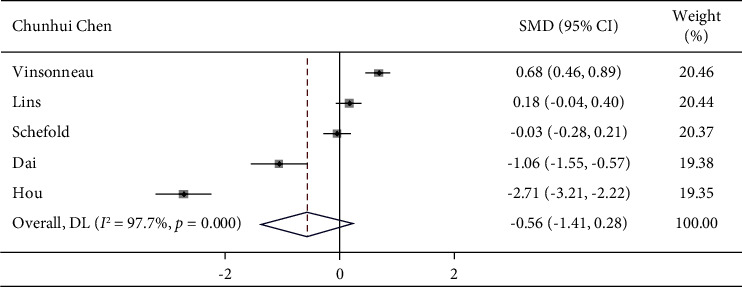
Forest plot of standardized mean difference and 95% CI of length of in-hospital stay. SMD: standardized mean difference; 95% CI: 95% confidence interval; ICU: intensive care unit. Note: Weights are from random-effects model.

Finally, we compared the length of in-hospital stay of patients who underwent different treatment strategies. The data were obtained from 5 studies. The results of meta-analysis showed that the length of in-hospital stay was significantly shorter in the CRRT group than that of the IHD group (RR: −0.56, 95% CI: −1.41-0.28, *P* < 0.05; I^2^ = 97.7%, Figure 4), which was similar to the results of length of ICU stay. We did not find significant publication bias of these studies according to the funnel plot (Supplementary Figure 5).

## 4. Discussion

In this study, we found that the CRRT had similar efficacy in renal recovery and short-term mortality compared with that of IHD in patients with renal failure in ICU. However, patients who underwent CRRT showed significant shorter length of ICU stay and in-hospital stay than that of patients received IHD. It highlighted the advantage of CRRT in ICU patients with renal failure.

AKI is one of the complications of critically ill patients, with high morbidity and mortality [24]. The main causes of AKI include sepsis, liver failure, cardiac surgery, hypovolemia, and antibiotic-related diseases. Clinically, it mainly manifests as elevated serum creatinine and decreased glomerular filtration rate, and the disease condition develops rapidly. Severe patients will cause anemia, nitrogen retention, dyspnea, if not promptly treated may endanger the patient's life [25]. Especially for patients with severe AKI, the prognosis is relatively poor due to the unstable hemodynamic status, high catabolism, and high-volume load [26]. In recent years, the incidence of AKI has been increasingly elevated, but there is no reliable clinical treatment. So, it is of great clinical significance to explore effective and appropriate treatment strategies for patients with renal failure in hospitals, especially in the ICU.

Currently, blood purification is usually used to remove toxins and inflammatory substances in patients and to stabilize the cell structure, microenvironment, and function to achieve the purpose of treatment [27]. At present, currently commonly used blood purification techniques for the treatment of AKI include IHD and CRRT [28]. IHD removes toxic substances such as toxins and inflammatory mediators from the patient's blood mainly by diffusion and convection, thereby purifying the blood and keeping the internal environment in a stable state [29]. However, the disadvantages of IHD include volume overload, hypercatabolism, and hemodynamic stability. If the patient is not effectively treated, ascites, cerebral edema, pleural effusion, and hyponatremia are likely to occur [30]. Systemic diseases such as heart failure and gastrointestinal bleeding will seriously aggravate the patient's condition and even adversely affect the patient's life. For patients with severe acute renal failure, it is usually accompanied by organ dysfunction, coupled with changes in the patient's metabolic function, which is easy to produce overload conditions [31]. Therefore, it is important to explore an effective and appropriate way to treat acute renal failure in clinical practice, optimize the adverse symptoms of patients, and improve the treatment effect. In recent years, CRRT has been widely used in clinical practice due to its advantages of stable hemodynamics, high solute clearance, and good biocompatibility. CRRT can continuously and slowly remove solutes in the body through adsorption, diffusion, and convection and can replace the fluid daily, of which the clearance rate is higher, helping to reduce the patient's systemic inflammatory response and improve the patient's immune function [32]. It can also provide nutritional support, stabilize the internal environment of the patient's body, and improve the prognosis of the patient. Studies have shown that CRRT treatment can remove inflammatory factors and reduce organ damage, thereby better maintaining the stability of vascular and intracellular and extracellular ion levels, acid-base balance, and osmotic pressure [33]. CRRT is therefore a promising technique to achieve better clinical outcomes. Our pooled results showed that CRRT showed similar efficacy in improving renal function and short-term mortality in patients with renal failure, further confirming the important role of CRRT in the treatment of renal impairment.

ICU is a place where modern medical theory and high-tech modern equipment were applied to conduct specialized and centralized monitoring, treatment, and nursing for critically ill patients [34]. Most of the patients in the ICU are in serious disease conditions. If patients are not treated appropriately, the length of hospital stay is prolonged and the incidence of complications increases. CRRT is widely used in clinical practice, especially in the ICU [35]. It can maintain the stability of hemodynamic parameters and improve the clinical symptoms and organ function of patients, which are important for reducing complications. In this study, we found that CRRT shortened the length of ICU stay and in-hospital stay in patients with renal failure, which has social and individual significance for reducing the heavy economic burden of renal failure.

### 4.1. Limitations

This study has several limitations. First, the number of included studies for analysis of renal recovery, length of ICU stay, and length of in-hospital stay is relatively small. More RCTs should be conducted in the future to further investigate the efficacy of these two strategies. Secondly, due to the lack of relevant data, we only investigated the health economic indicators including length of ICU stay and in-hospital stay. If sufficient data are available, other economic indicators such as the cost of illness should be analyzed. Thirdly, since the included studies for the renal recovery, length of ICU stay, and in-hospital stay are rare, it is difficult to precisely assess the publication bias of these studies. In-depth investigations with more studies are needed to further confirm the conclusions of this study.

## 5. Conclusion

Our comprehensive systematic review including 11 RCTs provides the available information on two common interventions to combat the renal failure in the ICU ward. Compared with IHD, CRRT had similar effects on renal recovery and short-term mortality in patients with renal failure in ICU. As a promising technique in clinical practice, CRRT could significantly reduce the length of ICU stay and in-hospital stay of patients, which was of great significance for the reduction of medical costs and the long-term benefits of patients, thereby reducing the burden on society and individuals.

## Figures and Tables

**Table 1:** Baseline characteristics of included studies.

Study	Study type	N	Age Sex (F%)	Disease	Design	Primary outcome	Secondary outcome	Risk
John, 2001, Germany	RCT	30	61 23%	ARF	IHD (lasting 3-4 h with blood flow rate 200-250 mL/min and dialysate flow rate 500 mL/min); *n* = 20 CVVH (18 mL/kg/h, and then 35 mL/kg/h) + Heparin; *n* = 10	In contrast to IHD, CVVH caused a decrease in heart rate (−3 (11) vs. 9 (8)/min, *P* < 0.01)) and an increase in systolic blood pressure (12 (1) vs. −5 (17) mmHg, *P* < 0.05) after 2 h	After 24 h, increased systemic vascular resistance was found in the CVVH vs. IHD group (312 (755) vs. −29 (89) dyne/cm^5^, *P* < 0.05) and there was a decrease in cardiac output (−1.54 (1.4) vs. −0.25 (0.91/min, *P* < 0.01)	L

Mehta, 2001, US	RCT	166	61 24%	ARF	IHD (flow rates of 500 mL/min, and blood flow rates of 200 to 300 mL/min, 2 sections, 3-4 h) + Heparin; *n* = 82 CAVHDF (blood flow rates of 100 mL/min, dialysate flow rates of 16.7 mL/min, and ultrafiltration rates of 400 to 800 mL/hour, >25 h) + Heparin; *n* = 84	The adjusted odds of death associated with CRRT was 1.58 (95% CI 0.7 to 3.3); overall, 36.6% patients (70.7% of those who survived) had a complete recovery of renal function, and there was no difference between the two groups (34.9% in CRRT vs. 33.3% in IHD, *P*=NS)	Days in ICU:(CRRT 15.1 vs. IHD 16.7 days, *P*=NS). Days in hospital were significantly reduced for patients who received CRRT as the initial therapy only (CRRT 17.1 vs. IHD 26.3 days, *P* < 0.01)	M

Gasparovic, 2003, Croatia	RCT	104	NR	ARF	IHD (lasting 3-4 h with blood flow rate 200-250 mL/min and dialysate flow rate 500 mL/min); *n* = 52 CVVH (18–35 mL/kg/h) + Heparin; *n* = 52	In the IHD group, survivors are 21; in the CRRT group, survivors are 15. There was no difference in total survival rate between the two groups	There were no differences between the groups in extracorporeal procedure	H

Augustine, 2004, US	RCT	80	61 32%	ARF	IHD (3 treatments weekly using a blood flow rate of 300 mL/min and dialysate flow rate of 500 mL/min) + Heparin; *n* = 40 CVVH (a blood flow rate of 200 mL/min) + Heparin; *n* = 40	There were no differences in survival or renal recovery between the groups. In patients who died, mean survival time was 10.7 (11.2) days for the IHD group versus 14.3 (16.1) days for the CVVH group	Death occurred in 27 CVVH patients (67.5%) versus 28 IHD patients (70%; =NS). Five patients on CVVH had renal recovery versus 4 on IHD (*P*=NS)	M

Uehlinger, 2005, Switzerland	RCT	125	67 31%	ARF	IHD (blood flow ranged from 150 to 350 ml/min, lasted from 3 to 4 h); *n* = 55 CVVH (blood flow ranged from 100 to 180 ml/min + heparin); *n* = 70	Mortality rates in the hospital (47 vs. 51%, CVVH vs. IHD, *P*=0.72) or in the ICU (34 vs. 38%, *P*=0.71). The percentage of patients with full recovery of renal function was 50% in the CVVH and 42% in the IHD group (*P*=0.61)	Hospital length of stay in the survivors on CVVH [20 (6-71) days] and in those on IHD [30 (2-89) days, *P*=0.25]. SCr: IHD 153 (59-496) vs. CVVH 121 (72-242) *μ*mol/L	M

Noble, 2006, UK	RCT	117	53 29%	ARF	IHD (4 h daily with a cuprophane membrane and heparin); *n* = 53 CHDF (a biocompatible membrane and prostacyclin in addition to heparin, 30 h); *n* = 64	There was no difference in ICU mortality (73.5% [39/53] IHD vs. 71.8% [46/64] CHDF, *P*=NS) or hospital mortality (83%[44/53] IHD vs. 76.5% [49/64] CHDF, *P*=NS) between the two RRT treatment groups	SCr: IHD 412.5 (70-966) vs. CHDF 345 (70-2240) *μ*mol/L; renal function at RRT initiation SCr: IHD 595 (260-1650) vs. CHDF 570 (200-2270)	M

Vinsonneau, 2006, France	RCT	360	65 27%	ARF	IHD (blood flow of 250 mL/min or more and dialysate flow set at 500 mL/min, 5.2 h); *n* = 184 CVVH (blood flow of 120 mL/min, dialysate flow of 500 mL/h, and ultrafiltration flow of 1000 mL/h); *n* = 175	Rate of survival at 60-days did not differ between the groups (32% in the intermittent hemodialysis group versus 33% in the continuous renal replacement therapy group (95% CI −8·8 to 11·1,), or at any other time	Days in ICU: IHD 20 (16-23) vs. CRRT 19 (15-22); days in hospital: 30 (24-35) vs. 32 (22-42) SCr: IHD 2.18 (1.8) vs. CVVH 2.12 (1.7) mg/dl	L

Lins, 2009, Belgium	RCT	316	66 41%	AKI	IRRT (4-6 h per session with a blood flow of 100-300 mL/min and a dialysate flow of 300-500 mL/min, 4 h); *n* = 144 CRRT (CVVH a blood flow rate of 100-250 mL/min and an ultrafiltration rate of 1-2 L/h, 4d) + anticoagulation; *n* = 172	A mortality of 62.5% in IRRT compared to 58.1% in CRRT. Days in ICU: 17.2 (18.7) vs. 18.7 (19.0); days in hospital: 31.4 (29.7) vs. 36.8 (31.0)	Mean SCr was 3.6 mg/dL in IRRT and 3.4 mg/dL in CRRT patients	M

Schefold, 2014, Germany	RCT	252	62 37%	ARF	IHD (4 hours of hemodialysis at a blood flow of 200 to 250 ml/min); *n* = 129 CVVH (24 hours daily by using a polysulfone synthetic membrane, blood flow of 200 ml/min); *n* = 123	Survival rates at 14 days after RRT were 39.5% (IHD) versus 43.9% (CVVH) (odds ratio (OR), 0.84; 95% CI, 0.49 to 1.41; *P*=0.50). 14 day-, 30-day, and all-cause intrahospital mortality rates were not different between the two groups	Days in ICU: IHD 25.2 (40.1) vs. CVVH 22.3 (26.1); days in hospital: 33.9 (49.3) vs. 32.4 (37.4) SCr: IHD 4.7 (0.8-10.9) vs. CVVH 3.9 (0.8-25.3) mg/dl	M

Dai, 2016, China	RCT	73	59 40%	AKI	IHD (blood flow of 150-200 mL/min or more and dialysate flow set at 500-1000 mL/min, 4 h) + heparin; *n* = 38 CVVH (blood flow of 150-200 mL/min and dialysate flow of 200-300 mL/h, 8-10 h) + heparin; *n* = 35	One week after treatment, compared to the IHD group, CRRT could dramatically reduce the levels of CRP (mg/L: 41.05 ± 10.15 vs. 60.21 ± 14.78, *t* = 6.401, *P* < 0.001), SCr (*μ*mol/L: 185.97 ± 65.48 vs. 232.02 ± 71.93, *P*=0.006), and urine output recovery time (days: 7.94 ± 3.06 vs. 11.08 ± 3.71, *P* < 0.001)	Length of ICU stay (days: 9.54 ± 3.39 vs. 13.42 ± 3.89, *t* = 4.521, *P* < 0.001), organ support time (days: 3.23 ± 2.70 vs. 6.34 ± 3.36, *t* = 4.343, *P* < 0.001), and the incidence of cardiovascular events (23.53% (8/35) vs. 39.47% (15/38), *χ*2 = 5.509, *P*=0.025)	L

Hou, 2021, China	RCT	120	55 28%	AKI	IHD (blood flow of 220-250 mL/min and dialysate flow set at 500 mL/min, 6 h) + heparin; *n* = 60 CVVH (blood flow of 120-200 mL/min and dialysate flow of 25-45 mL/kg/h, 24 h) + heparin; *n* = 60	In the patients treated with CRRT, the levels of SCr, blood urea nitrogen, and blood lactic acid were all lower than those in the control group (all *P* < 0.05)	The time of staying in ICU, the period of oliguria, the time of renal replacement therapy, the time of staying in hospital, and the experimental group were all shorter than the control group (all *P* < 0.05)	M

Acute kidney injury (AKI); acute renal failure (ARF); continuous arteriovenous hemofiltration (CAVH); continuous arteriovenous hemofiltration with dialysis (CAVHD); continuous arteriovenous hemodiafiltration (CAVHDF); continuous arteriovenous hemofiltration (CAVHF); Continuous hemodialysis with ultrafiltration (CHDF); continuous venovenous hemofiltration (CVVH); continuous renal replacement therapy (CRRT); intermittent hemodialysis (IHD); intermittent renal replacement therapy (IRRT); randomized clinical trial (RCT); serum creatinine (SCr).

## Data Availability

The datasets in this study are available from the corresponding author on reasonable request.

## Supplementary Materials

Supplementary Materials Supplementary Figure 1. Risk of bias of the included studies with the tools provided by Cochrane. Supplementary Figure 2. Funnel plot for the evaluation of publication bias of studies comparing the renal recovery. Supplementary Figure 3. Funnel plot for the evaluation of publication bias of studies comparing the short-term mortality. Supplementary Figure 4. Funnel plot for the evaluation of publication bias of studies comparing the length of ICU stay. Supplementary Figure 5. Funnel plot for the evaluation of publication bias of studies comparing the length of in-hospital stay.
