# Supplementary material for: Efficacy of Continuous Renal Replacement Therapy and Intermittent Hemodialysis in Patients with Renal Failure in Intensive Care Unit: A Systemic Review and Meta-analysis
Source: Evid Based Complement Alternat Med. 2023 Apr 17;2023:8688974. doi: 10.1155/2023/8688974 (PMC10125730; doi:10.1155/2023/8688974)
Supplement: Supplementary Materials — Supplementary Figure 1. Risk of bias of the included studies with the tools provided by Cochrane. Supplementary Figure 2. Funnel plot for the evaluation of publication bias of studies comparing the renal recovery. Supplementary Figure 3. Funnel plot for the evaluation of publication bias of studies comparing the short-term mortality. Supplementary Figure 4. Funnel plot for the evaluation of publication bias of studies comparing the length of ICU stay. Supplementary Figure 5. Funnel plot for the evaluation of publication bias of studies comparing the length of in-hospital stay. [file 8688974.f1.docx]

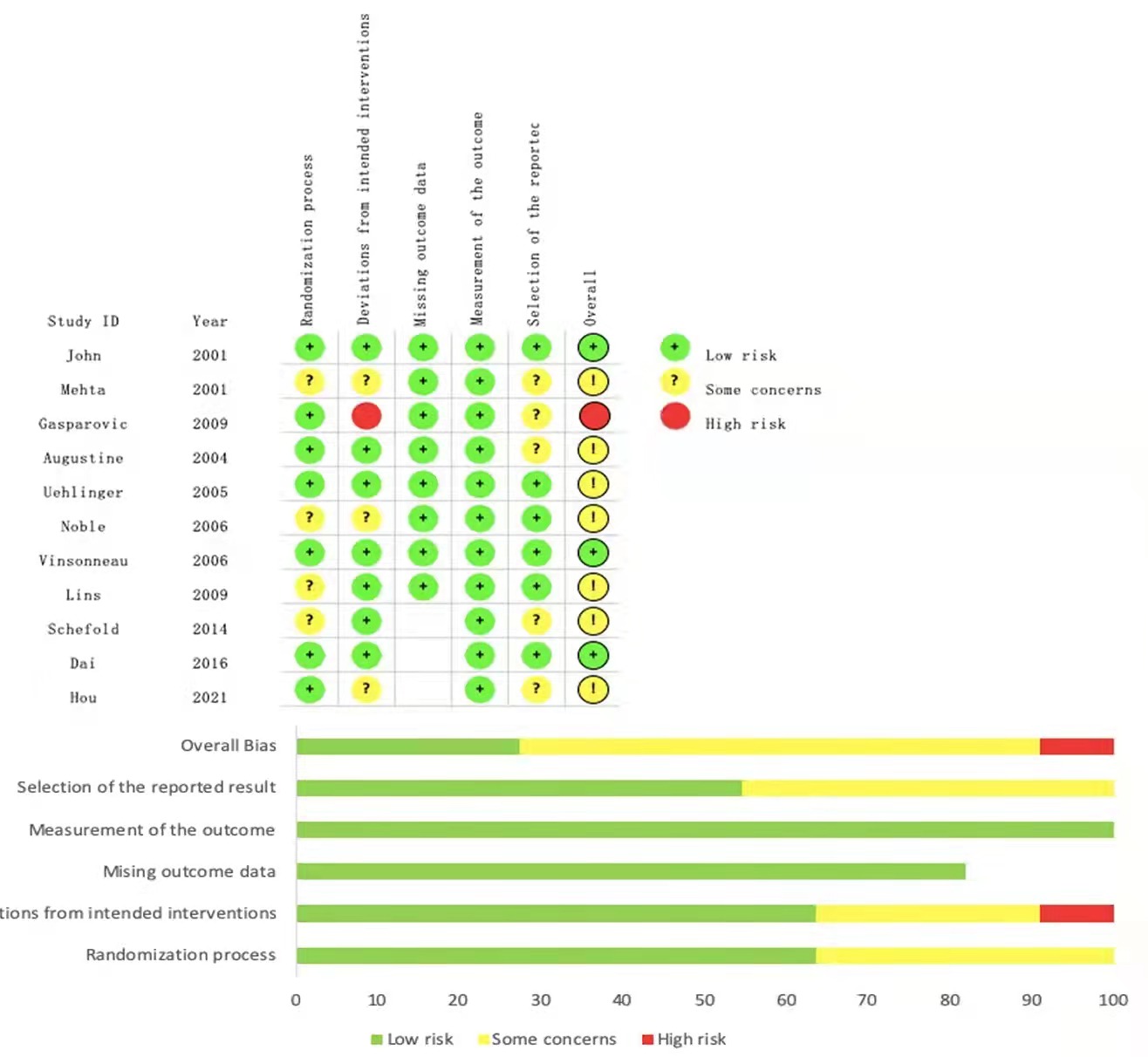


**Supplementary figure 1.** Risk of bias of the included studies with the tools provided by Cochrane.


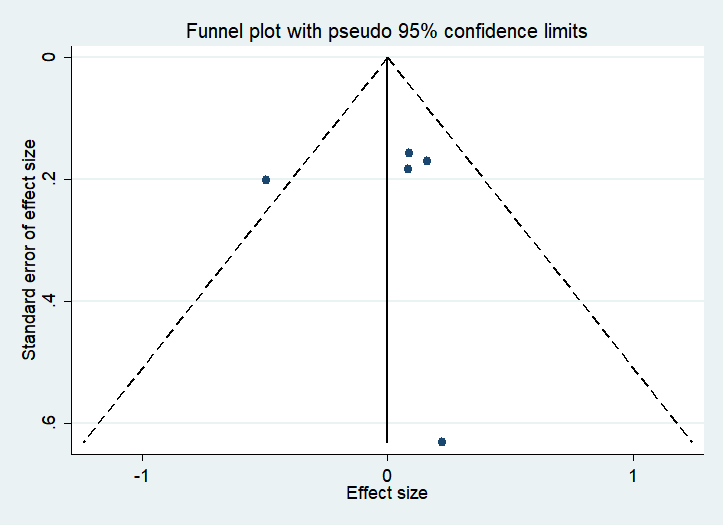


**Supplementary figure 2.** Funnel plot for the evaluation of publication bias of studies comparing the renal recovery.


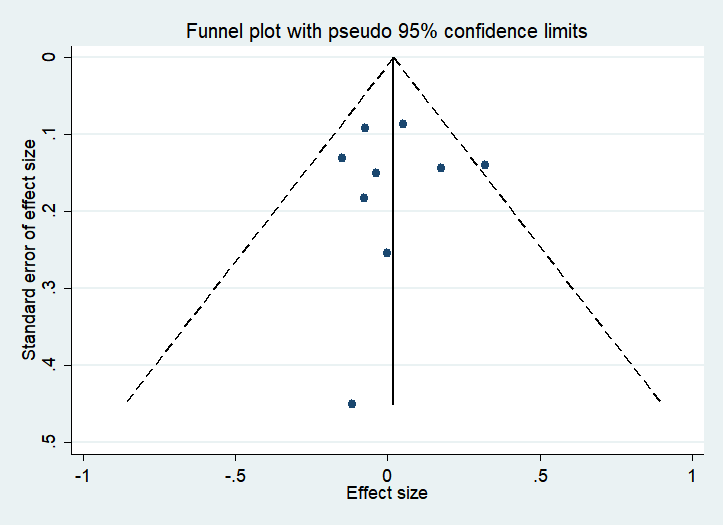


**Supplementary figure 3.** Funnel plot for the evaluation of publication bias of studies comparing the short-term mortality.


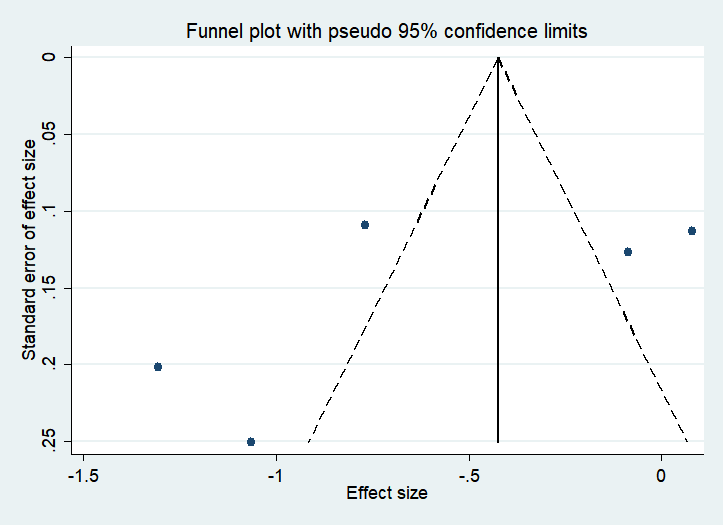


**Supplementary figure 4.** Funnel plot for the evaluation of publication bias of studies comparing the length of ICU stay.


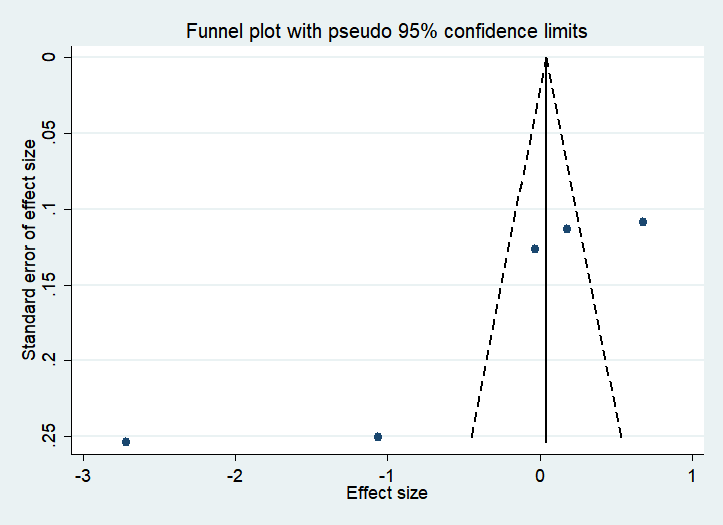


**Supplementary figure 5.** Funnel plot for the evaluation of publication bias of studies comparing the length of in-hospital stay
